# Supplementary material for: Are carbon emissions trading and green financial instruments synergistic? -Comprehensive quantitative research based on content analysis
Source: PLoS One. 2024 Mar 7;19(3):e0298601. doi: 10.1371/journal.pone.0298601 (PMC10919601; doi:10.1371/journal.pone.0298601)
Supplement: S1 Data — (ZIP) [file pone.0298601.s001.zip › Data/地级市Z_F工作报告.zip!/╔╜╢1⁄2/╘μ╫»╩╨/└┤╫╘╣1⁄2╓┌║┼í╛┬φ┐╦╩2╛▌═°í┐_2014.docx]

各位代表：

现在，我代表市人民政府向大会作政府工作报告，请予审议，并请市政协委员和其他列席会议的同志提出意见。

一、2013年政府工作回顾

2013年是宏观经济形势较为复杂、经济下行压力加大、转型发展很不平凡的一年。一年来，全市上下在市委的正确领导下，坚定不移地实施城市转型战略，抢抓机遇，奋力攻坚，经济社会发展实现了稳中有进、稳中向好，提速转型、跨越发展迈出了坚实步伐。全市GDP完成1830.6亿元，增长10.1%；地方财政收入完成130.7亿元，增长12.3%，扣除省级下划部分可比口径增长6.4%；固定资产投资完成1238亿元，增长20.5%；社会消费品零售总额达到627亿元，增长13.5%；外贸进出口总额达到12.5亿美元，增长10.7%；实际利用境外资金1.6亿美元，增长11.3%；城镇居民人均可支配收入25238元，增长9.9%；农民人均纯收入10878元，增长13.2%，连续4年高于城镇居民收入增幅。

(一)工业转型步伐加快。坚定不移地推进市属工业企业改革，2013年启动实施了33家企业改革改制和社会化管理，2万余名困难职工的历史遗留问题基本得到解决，市南工业区转入以基础设施建设、棚户区改造、产业招商为主的发展阶段。着力推进传统产业市场化重组，泉兴中联覆盖全市三分之二以上水泥产能，促成了中联水泥集团运营总部落户枣庄；八一轮胎成功重组丰源轮胎；泉兴集团实现对金庄煤矿的托管。持续推进产业改造升级，实施工业技改项目516个，完成投资267亿元。锂电自行车、润恒光伏、华润三九医药、鲁南装备制造等“四新”产业项目建设顺利，高新技术产业产值占规模以上工业总产值的比重提高到17.4%。大力扶持中小微企业发展，滚动拨付过桥还贷资金5.2亿元。以更大的力度抓招商引资，引进日本JFE、美国喜达屋等世界500强企业投资项目10个。百个重点项目建设完成投资372亿元，薛城能源循环经济产业园、十电2×60万千瓦机组等一批大项目开工建设，联想百万吨烯烃、辰阳数控机床等项目进展顺利。全市在建投资亿元以上项目303个，其中过10亿元项目63个。

(二)服务业发展亮点增多。以服务业重点项目建设为抓手，促进服务业提档升级。服务业固定资产投资完成635亿元，增长21.7%，占固定资产投资比重达51.3%。台儿庄古城重建全面竣工，省人大常委会通过保护条例；古城文化产业园、非遗博览园规划建设继续推进。微山湖古镇一期建成开放，冠世榴园、抱犊崮、熊耳山等重点景区服务功能进一步完善，成功创建为省乡村旅游示范市。全市旅游综合收入111.6亿元，增长16.5%。大力培植高端服务业，上海跨采中心枣庄分中心、泰国世博城、香港天合数码城、鲁南之家电子商务等项目签约落地，数据中心、万洲商贸、义乌商城等项目建设顺利，红星美凯龙、居然之家、大润发等知名品牌落户枣庄。坚持政府引导、市场运作，成功举办年博会、世界石榴大会、国际机床工具展览会、国际锂电产业博览会等节会活动。引进软件和服务外包企业11家。济宁银行枣庄分行挂牌营业，青岛银行枣庄分行获批筹建，枣庄农商行组建加快推进，32家企业实现股权挂牌托管。

(三)农村改革纵深推进。加快国家农村改革试验区和现代农业示范区建设，积极推进农业产业化、农村城镇化、农民市民化。深化农村土地使用产权制度改革，新增土地合作社492家、流转土地19.7万亩、农地抵押贷款1.9亿元，分别增长68%、37%和62%。注册成立全省首家农村产权交易中心，农村集体土地所有权确权登记发证全面完成，家庭农场、规模以上龙头企业分别发展到481家和288家。新创建农业标准化生产基地3.8万亩，新认证“三品一标”农产品45个，农产品质量抽检合格率达到98.1%。完成农田水利建设投资12亿元。农业综合机械化水平达到80%，农作物良种覆盖率达到98%。落实农业“四补一保”资金4.5亿元。百个美丽乡村创建扎实开展，3个村被列为国家千村试点。大力推进城乡环卫一体化，目前已覆盖半数以上村居。

(四)城镇化进程全面提速。坚持四轮驱动、四级联动，着力优化布局、突出特色、完善功能，不断提升城镇化质量。完成城市总体规划修编和示范镇规划编制，基本建立了城乡一体的规划体系。出台加强城市管理30条意见，城市规划建设管理水平进一步提升。统筹推进城市基础设施建设，完成投资46亿元，增长10.3%。新城实验高中一期、龙潭公园建成启用，实验小学、市妇保院新院、市立医院新院、文体中心等项目进展顺利，11处便民商业网点投入使用。新建、改造城市主次干道86条，新建污水管网82公里，区(市)城区承载能力进一步提升。强力推进中心城区集中供热改造，新建供热一级管网47公里，新增供热面积538万平方米。以示范镇为引领加快小城镇发展，完成小城镇建设投资35亿元、增长8%，新争取7个镇纳入省级示范镇。稳步推进农村新型社区建设，新建农村社区服务中心100个，改造农村危房2672户。深入开展城乡环境综合整治，顺利通过省级卫生城市复审。开通薛城至陶庄的BRT，改造城乡客运一体化线路6条。争取枣鱼高速公路东延，完成104国道改建、枣济线改造，新建、改造农村公路868公里，城乡路网结构进一步优化。

(五)生态建设初见成效。全面完成中心城区南部山系绿道建设、枣临高速和抱犊崮旅游大道绿化，扩种石榴3.7万亩，新增造林绿化面积26万亩，环城森林公园绿道总长达到190公里，市域森林覆盖率达到34%以上。实施重点节能项目50个，预计万元GDP能耗降幅完成省下达任务。关闭30万吨以下小煤矿9处、采石场25处、储煤场150处，城市扬尘、机动车尾气、工业废气治理和秸秆禁烧力度进一步加大，蓝天白云天数达到144天。深入开展南水北调和淮河流域水污染防治，重点治污工程建设完成80%以上，境内7条主要河流实现鱼类稳定生长。

(六)民生保障更加有力。百件惠民实事得到较好落实，民生支出占财政总支出比重达到53%。新增城镇就业6万人，农村劳动力转移就业7万人。城市低保、城乡居民基础养老金发放标准分别提高到每人每月360元和70元，农村低保、五保集中和分散供养标准分别提高到每人每年2200元、4000元和2700元，企业退休人员基本养老金实现“九连增”。新建社区老年人日间照料中心32处、农村幸福院100处。开工建设保障性住房1890套，竣工1005套。新建幼儿园35所，新建、维修校舍31万平方米。医药卫生体制改革稳步推进。人口自然增长率控制在5.7‰以内。成功承办十艺节民乐展演。市八运会圆满落幕。物价总水平保持基本稳定。平安枣庄建设扎实推进，安全生产事故起数、死亡人数实现“双下降”。其他社会事业也都取得了新发展。

(七)发展环境持续优化。严格执行中央“八项规定”，坚决解决“四风”问题。厉行勤俭节约，市级“三公”经费和会议费下降26%。自觉接受人大、政协监督，566件人大代表建议和政协提案全部按时办结。充分发挥市长热线、市长信箱作用，受理群众来电来信2.7万件，办结率99%。深化行政审批制度改革，市级行政许可事项取消30项、下放78项、调整25项、保留110项，成为目前全省行政许可事项最少的市。大力精减政府文件和议事机构，政府普发性公文同比减少33%；清理政府议事机构139个，保留71个。扎实开展“庸懒散”专项治理、“百千百”民主评议和社情民意调查，不断强化网上电子监察和日常审计监督，从严查处涉企乱收费等行为，营商环境进一步优化。

各位代表，一年来，在省委、省政府和市委的坚强领导下，在人大、政协和社会各界的监督支持下，在全市人民的共同努力下，我们经受住复杂局面的考验，取得了来之不易的成果。在此，我代表市人民政府，向奋斗在各行各业的广大干部群众，向解放军、武警驻枣官兵和中央、省驻枣单位，向在我市创业发展的投资者、建设者，向所有关心、支持枣庄发展的港澳台同胞和海内外朋友，表示衷心的感谢和崇高的敬意!

同时，我们也应看到发展中存在的困难和问题：我市经济总量依然较小，缺乏带动作用强的大企业、大项目；产业结构偏重、质效偏低的问题仍然存在，传统产业产能过剩，新兴产业支撑不足，服务业发展水平亟待提升，结构调整任重道远；城镇化质量、农业产业化水平不高，统筹城乡发展的任务依然艰巨；资源、环境等要素制约比较突出，市场化配置机制尚未完全形成；教育、医疗等基本公共服务供给不够均等，解决就业、社保等民生难题还要付出更大努力；政府职能转变还不够到位，优化营商环境的力度仍需加大，等等。对于这些问题，我们一定高度重视，以改革创新的思路，制定切实可行的措施，尽快加以解决。

二、2014年政府工作目标和总体要求

2014年是全面贯彻落实党的十八届三中全会精神的开局之年，是枣庄提速转型、跨越发展的关键之年。做好今年的政府工作，任务艰巨，责任重大。我们必须更加清醒地认识到，当前全球经济进入深度调整期，国内经济企稳回升的基础尚不稳固，加速转调创面临诸多挑战。党的十八届三中全会吹响了全面深化改革的号角，省委、省政府拉开了建设西部经济隆起带的序幕，枣庄这座老工业基地踏上了奋起赶超、重塑辉煌的新征程。挑战和机遇并存，困难与希望同在。我们必须更加准确地把握面临的机遇，更加自觉地担当起历史的重任，积极进取，务实作为，用实干托起枣庄人民的振兴梦。

今年政府工作的总体要求是：深入贯彻落实党的十八大和十八届二中、三中全会精神，牢牢把握西部经济隆起带建设的重要机遇，以稳中求进为总基调，以改革创新为动力，以提高经济发展质量和效益为中心，以转方式调结构为主线，以保障和改善民生为目的，坚持项目兴市、工业强市、生态立市，坚持四化同进、四城同创，纵深推进三大战役，倾力打造转型升级和经济文化融合发展高地。

今年经济社会发展的主要预期目标是：全市GDP增长10%左右；地方财政收入增长10%左右；固定资产投资增长18%；社会消费品零售总额增长13%；外贸进出口总额增长5%；城镇居民人均可支配收入、农民人均纯收入分别增长11%和12%；城镇登记失业率控制在3.8%以内；人口自然增长率控制在9.6‰以内；居民消费价格涨幅控制在3.5%以内；全面完成省下达的节能减排任务。

实现上述目标，必须统筹把握好以下五个方面：一是必须把稳增长促转调作为首要任务。城市转型遭遇经济下行，倒逼我们要强化底线思维，注重危中寻机、难中求进，以破釜沉舟、背水一战的决心，以凤凰涅槃、浴火重生的胆识，加快转调步伐，加速转型振兴，实现有质效、可持续的稳定增长。二是必须把抓改革解难题作为关键环节。面对制约发展的深层次问题，以新的理念谋划工作，勇于担当，不回避、不犹豫、不退缩，果断决策，精准发力，持之以恒打好改革组合拳。三是必须把守底线惠民生作为最大责任。坚持从群众不满意的地方改起，从群众最期盼的事情做起，量力而行、尽力而为、统筹城乡、兼顾各方，将有限的财力更多地向民生领域倾斜，扎实办好惠民实事，不断提升群众的满意度、幸福感。四是必须把打基础利长远作为重要遵循。不贪眼前之利，不求一时之快，多上符合科学发展要求的大项目、好项目，为未来发展积蓄后劲；多筑桥修路、盖学校、建医院，为长远发展提供支撑；多植树种绿、减排治污、修复生态，为枣庄留下更多的青山绿水。五是必须把转作风促落实作为根本保障。一分部署，九分落实。思路再好、政策再优、措施再多，落不到实处都是一纸空文。全市上下必须围绕既定的目标，把更多的精力用在抓落实上，出实招、用真功，以“钉钉子”的精神，一项一项地盯紧靠牢，一件一件地抓实办成，努力把枣庄发展的美好蓝图变为生动现实。

三、全面深化经济体制改革

改革是当今时代的主旋律，也是转方式调结构稳增长的最大动力。必须紧跟中央和省里的改革步伐，正确、准确、有序、协调推进各项改革，释放新一轮发展红利。

(一)继续深化行政审批制度改革。在承接好省里下放行政审批事项的基础上，再取消、下放一批行政审批事项，用政府权力的“减法”换取市场活力的“乘法”。全面落实优化营商环境30条措施，深化“两集中、两到位”，实行投资项目并联审批、联合勘验、无偿代办，完善审批“绿色通道”，为企业和群众提供高效便捷的审批服务。建成启用市、区(市)两级政务服务信息平台，实现信息共享、上下联动。推进工商注册制度便利化，允许各类市场主体平等进入负面清单之外领域。进一步清理规范涉企收费和各类检查评比，切实减轻企业负担。推动行业协会、中介组织与行政机关脱钩，引入竞争机制建立中介机构服务平台，加大政府向社会购买服务力度。

(二)继续深化农村改革。加快国家农村改革试验区建设，纵深推进农村土地使用产权制度改革，进一步扩大产权改革范围、新型农业经营主体数量、产权抵押贷款数额、农地确权登记面积、农村产权交易范围，建立完善农村产权交易体系、新型农业经营体系和社会化服务体系，土地合作社数量、流转土地面积、农地抵押贷款分别增长15%、10%和20%以上，农村土地承包经营权确权登记发证完成80%以上。加快农村宅基地确权登记发证，赋予农民更多的财产权，探索增加农民财产性收入的有效渠道。推进农村集体资产股权化改革，完善被征地农民利益保障机制。积极争取全国农村集体经营性建设用地流转试点，有序推进农村建设用地出让、租赁和入股。

(三)继续深化企业改革。坚定不移地实施市属工业企业三年改革攻坚计划，在巩固原有45家企业改革改制成果的基础上，今年新启动21家市属工业企业改革改制。加快市南工业区建设发展，统筹推进基础设施配套、棚户区改造和产业招商，重点抓好大世界基尼斯枣庄基地等项目建设。支持山东万泰等“退城进园”，让老企业焕发新的生机。进一步理顺国有资产监管体制，引进多种所有制主体参与国有企业改革。完善公司法人治理结构，推行职业经理人制度，探索开展岗位分红权激励试点，增强国有企业发展活力。利用过桥还贷资金等形式，扶持中小微企业健康成长。

(四)继续深化财税体制改革。全面推进预算绩效管理，建立权责匹配的财政支出制度，清理规范重点支出同财政收支增幅挂钩事项，加快形成“花钱必问效、无效必问责”的管理机制。扩大财政预决算、部门预决算及“三公”经费预决算公开试点范围，2015年实现全面公开。改进专项资金管理办法，逐步取消竞争性领域专项和基层资金配套，完善一般性转移支付增长机制。规范税收优惠政策，深入推进“营改增”试点，完善地方税征管体系。着力优化财政收入结构，进一步提高财政收入质量。积极探索国有资本投资运营新模式，提高国有资产经营效益。加强政府债务管理，规范政府举债融资行为，有效防范和化解债务风险。

(五)继续深化金融领域改革。大力引进异地金融机构，争取更多的股份制银行落户枣庄。做大做强本地金融机构，支持枣庄银行引入战略合作伙伴，实现枣庄农商行挂牌营业。探索开办涉农金融保险，鼓励创办村镇银行、土地银行，积极发展民间资本管理公司、农村资金互助社等新型金融组织，实现小额贷款公司区(市)全覆盖。支持企业跨界参与金融经营，加强与第三方融资平台的合作，有效对接互联网融资市场。推广区域集优集合票据、知识产权质押贷款、企业私募债、创投基金等金融产品，加快企业上市和“新三板”挂牌步伐，推进企业在区域资本市场挂牌托管，提高金融服务实体经济的能力。

(六)继续深化其他领域改革。健全工资正常增长机制，完善最低工资和工资支付保障制度。有序推进事业单位分类改革，完善动态考核和收入分配机制。大力实施学生体质健康提升和艺术教育普及计划，完善校长、教师交流轮岗制度，推动城区优质教育资源扩容。支持高等院校特色化办学，培养更多实用型人才。加快公立医院改革，探索建立现代医院管理制度，保障公立医院的公益性。整合城镇居民基本医疗保险和新型农村合作医疗，建立城乡医疗保险统一管理制度。探索机关事业单位社会保险制度改革，逐步建立基本养老保险和职业年金相结合的多层次养老保障体系。

四、加快调整产业结构

把枣庄打造成为转型升级和经济文化融合发展高地，关键是要调整偏重的产业结构，转变粗放的发展方式，促进传统产业优化升级，推进新兴产业加快崛起。转方式调结构促升级绝非一日之功，必须牢牢抓住项目建设这个牛鼻子，着力推动高端、高质、高效发展。

(一)加快新型工业化。坚持“工业强市”，完善产业发展规划，打好工业转型振兴攻坚战，实现工业经济的华丽转身。力争用5年左右的时间，培育壮大煤化工、煤电能源、机械机床3个千亿产业板块，新型建材、橡胶塑料、纺织服装、食品加工4个500亿产业板块，新能源、新医药、新材料、新信息和节能环保5个百亿产业板块；重点培育枣矿集团千亿企业集团，联想化工、盛隆、鲁化、泉兴等10个百亿企业集团，华电十电、华润纸业等10个50亿企业集团，形成多业支撑、竞相发展的良好局面。加大市场化重组力度，提高传统产业的集中度和竞争力。巩固水泥产业整合重组成果，发挥中联水泥集团运营总部的引领带动作用，提升行业经营管理水平。加强与山能集团的合作，加快八一丰源轮胎二期项目建设，年内形成1000万条子午线轮胎生产能力。引导鲁南机床、威达重工等龙头企业对外联合、对内重组，促进机床产业转型升级。加快联想烯烃、薛城能源等项目建设，大力发展烯烃、炭黑、针状焦等下游产品，提高煤化工产业发展质量和效益。促进工业化与信息化深度融合，推动传统产业技术革新、工艺升级、产品换代。抓好50个技改重点项目，力争全市工业技改投资增长15%以上。继续推进锂电自行车、锂电正极材料、锂电隔膜等项目建设，不断壮大锂电产业整体实力。扶持润恒光能等企业加快发展，着力推进500兆瓦光伏二期等项目建设，促进光伏产品的推广应用。抓好华润三九医药、威智医药、康力医疗器械产业园等重点项目，打造国内重要的医药产业基地。加快推进鲁南装备制造、中科蓝天地源热泵等项目建设，提高产品附加值和市场份额。突出企业主体地位，鼓励企业创建重点实验室、工程实验室、院士工作站等研发机构，引导大中型企业建立高水平研发平台。加强与高等院校、科研机构的战略合作，推进煤化工、机床、锂电等重点产业技术研究院建设，加快创新成果转化和产业化步伐。采取项目外包、人才租赁等柔性方式引进专业人才，为产业转型提供智力支持。强化环保、能耗、土地和矿产开发等指标约束，推行等量置换、减量置换，严格限制新增过剩产能。落实差别电价政策和资源环境保护税费制度，引导过剩产能有序退出。年内淘汰平拉工艺平板玻璃生产线、年产1万吨以下废纸制浆生产线和15年以上染整设备等落后产能。2015年底前全面关闭30万吨以下小煤矿和小石膏矿。

(二)加快服务业高端化。坚持龙头带动、品牌引领，促进服务业发展提速、比重提高、质量提升。加快台儿庄国家级文化产业园建设，丰富古城文化业态，依托非遗博览园、版权贸易基地等平台，吸引更多的文化产业项目和创意人才聚集。抓好中兴矿山公园、石榴文化博览园和聚艺谷等文化创意园建设，形成各具特色的文化产业聚集区。按照“一园三镇五点”的思路，全域化布局乡村旅游。以环中心城区绿道为纽带，将沿线景点串珠成链，科学布局休闲驿站、农家乐等要素，打造环城森林公园。以滨湖镇、榴园镇、北庄镇为重点，打造生态旅游特色镇。以台儿庄古城、微山湖古镇、冠世榴园、抱犊崮、熊耳山为节点，提升精细化管理和市场化营销水平，打造精品旅游景区。以民俗文化、观光采摘、娱乐健身、休闲养生为内容，着力培育“运河人家”“榴园人家”“山乡人家”“湿地渔家”等品牌，打造乡村旅游目的地。加快鲁南铁水联运、贵诚物流、德合物流等项目建设，发展临港物流、仓储物流、冷链物流，努力建设区域性物流枢纽。加快国际机床市场、汽车文化贸易产业园等项目建设，培育区域性商品集散中心。抓好跨采中心枣庄分中心、泰国世博城等项目建设，打造城市核心商圈。改造提升一批传统商业街区，整治规范一批特色餐饮街区，引进一批高端百货、品牌连锁店，优化城区商业网点布局。鼓励社会力量创办老年康复保健中心、健康体检中心、老年公寓等服务机构，促进健康养生产业发展。紧跟大数据和互联网发展步伐，以云计算、物联网、4G网络为支撑，加快“智慧枣庄”建设。着力培育台儿庄软件和服务外包基地、鲁南信息产业园，做大同诚卡、鲁南之家等电子商务平台。市场化办好年博会、机床产业博览会、锂电产业博览会等节会活动，带动会展交易和社会消费。

(三)加快农业现代化。大力发展优质高效特色农业，抓好石榴、马铃薯等10大特色农业示范基地，培育一批现代农业示范镇和“一村一品”专业村。大力发展农产品精深加工，重点培育祥和乳业、春藤食品等10大龙头企业，新增省级重点龙头企业5家以上。大力发展订单农业，推广“市场+基地+农户”经营模式，提高农业生产经营效益。创新农产品流通方式，重点扶持杏花村市场、豆制品基地等10大农产品交易市场，规范提升100家农社对接直销店。抓好农业科技成果应用，加大中低产田改造、土壤改良修复、农业面源污染防治力度，积极推广现代种业、配方施肥、节水灌溉和农机化生产。大力发展有机、绿色、无公害农产品，新认证“三品一标”30个以上。推广标准化生产，抓好国家级食品安全检测中心、农产品综合质检中心项目建设，构建全过程农产品安全监管体系。认真落实“四补一保”惠农政策，加大对农业的财政和金融支持力度。加快岩马水库除险加固，继续推进庄里水库建设，进一步改善农业生产条件。稳步提升粮食、蔬菜生产能力，确保全市人均占有千斤粮、一吨菜。

(四)加快对外开放。瞄准国内外500强企业，开展产业招商、定点招商、登门招商，引进一批带动力强的大项目。发挥骨干企业的招商主体作用，通过合资合作、兼并重组等方式实现强强联合、借力发展。完善园区发展规划，引导项目向园区集中。创新园区投入方式，强化基础设施配套，为项目落地提供支撑。依托大企业、大项目，吸引上下游企业集聚，培育一批配套程度高、专业协作好、特色鲜明的高端产业集群和产业基地。清理批而未供、供而未用土地，盘活闲置土地资源，促进土地节约集约利用。健全以亩均投入、亩均产出、亩均税收、亩均就业人数为导向的项目考核、退出机制，促进园区“腾笼换鸟”“二次开发”。支持枣庄高新区、滕州经济开发区创建国家级园区。加大利用外资和招商考核力度，鼓励现有外资企业增资扩股、境外融资，把引资、引智、引技有机结合，拓展利用外资领域。注重以外资促外贸，加快培育出口导向型生产加工企业，做大做强机床、机电、服装、农副产品加工等外贸出口基地。继续实施“走出去”战略，鼓励有条件的企业到境外开发资源、开拓市场。巩固深化台湾·山东经贸文化周招商成果，依托海峡两岸交流基地，加强对台文化交流和经贸合作。

五、提高城镇化发展质量

新型城镇化的核心是人的城镇化。必须以满足人的需求、促进人的发展为目的，突出规划引领、生态优先、基础先行、产业支撑、功能完善、城乡统筹，科学、有序、稳妥推进新型城镇化。

(一)统筹推进城乡规划建设管理。实施全域城镇化战略，编制好新型城镇化发展规划，切实做到“四级全覆盖、城乡一张图”。突出地域特色，创新规划理念，优化城镇化布局和形态，保护和弘扬优秀传统文化，让居民望得见山、看得见水，留住枣庄的发展印记。加强规划协同，搞好城镇规划与土地规划的衔接，提高城镇土地利用率，拓展城市发展空间。坚持市场导向，建立多元化、可持续的城乡建设资金保障机制。加强房地产市场调控和工程质量监管，促进建筑业健康有序发展。进一步理顺城市管理体制，实行市级统筹、属地负责、重心下移，建立大城管格局和长效监督考评机制，提高城市精细化管理水平。

(二)统筹推进中心城区和区(市)城区建设。围绕增强大新城的承接、辐射和带动功能，重点抓好实验小学、实验高中二期、职业中专等教育设施建设，市妇保院新院、市立医院新院等医疗设施建设，文体中心、图书大厦等文体设施建设，双子座、枣矿城等商住设施建设，东西绿轴、蟠龙河湿地等生态景观设施建设。围绕提升区(市)城区人居环境，建设一批保障性安居工程、绿色生态小区，大力推进主次干道、背街小巷、地下管网等基础设施改造，合理规划建设城市公交泊站、公共停车场、自行车道及步行道等设施，科学开发利用城市地下空间，多建为民利民的“贴心工程”“良心工程”，逐步形成布局合理、功能完备、安全可靠的基础支撑体系，让枣庄变得更加宜居。

(三)统筹推进小城镇和农村新型社区建设。坚持宜工则工、宜商则商、宜游则游，明确功能定位和产业布局，引导小城镇特色化发展。对示范镇赋予更多的权限、给予更多的支持，加快示范镇建设发展。每个示范镇争取建设一处自来水厂、一座污水处理厂、一座垃圾中转站、一个集贸市场、一处公园绿地、一条特色街区、一个示范社区、一个支柱产业。建立城乡统一的户口登记制度，优先解决在城镇稳定就业的农业人口落户问题，加快实现社保、教育、医疗等基本公共服务覆盖城镇常住人口。有序开展空心村、城郊村治理，加大薄弱村、偏远村帮扶力度，抓好农村新型社区建设，新建农村社区服务中心60个，改造农村危房1500户，让枣庄变得更加美丽。

(四)统筹推进生态枣庄建设。坚持“生态立市”不动摇，大力实施中心城区环城绿道建设、城市绿化提升、荒山绿化、石榴园扩建、东沙河综合整治等创森重点工程，抓好城区裸露土地覆绿和建筑立体绿化。年内新增造林绿化面积13万亩，市域森林覆盖率、城市绿化覆盖率分别达到35%和40%以上，力争创建成为国家森林城市。启动国家园林城市创建工作，力争2015年创建成功。严格控制露天矿山开采，加大矿山地质环境和破损山体治理修复力度，建设绿色矿山。探索建立节能量市场化交易机制，全面推进重点领域节能减排，完成重点企业脱硫脱硝治理任务。加强工业废气、城市扬尘、机动车尾气综合治理，继续抓好秸秆禁烧和综合利用，稳步推进建成区锅炉“煤改气”，巩固治超治限成果，加快淘汰“黄标车”，努力在雾霾治理方面取得实效。健全“治、用、保”流域综合治污体系，抓好水污染防治重点项目建设，坚决打赢迎淮核查攻坚战，持续改善流域水环境，让枣庄变得更加环保。

(五)统筹推进城乡交通一体化。抓好十大交通工程，建成启用客运换乘中心，启动枣鱼高速公路东延、高铁换乘枢纽建设和北留线改建，做好沂台线南延、滕薛线改建、万年闸复线船闸工程前期工作，加快沿运港口和航道升级改造，争取枣庄机场列入国家“十三五”专项规划；抓好中心城区道路建设改造，进一步优化城市公交网络，实现城区公共自行车服务全覆盖；继续抓好农村公路新建改造和养护管理，推进城市公交向镇村延伸，努力构建城乡互通、高速绕城、公铁水联运的综合交通网络，让枣庄变得更加通畅。

(六)统筹推进城乡环境整治。持续开展城市环境综合整治提升活动，重点整治城乡结合部、老旧小区、背街小巷、农贸市场等领域，规范城区摆摊设点、车辆停放、户外广告设置。继续推进百个美丽乡村创建，实施村容村貌整治和改厨改厕，改善农村环境。完善“户收集、村集中、镇转运、区(市)处理”的运作模式，健全垃圾收集处理、组织管理、经费保障、检查考核体系，上半年实现城乡环卫一体化全覆盖。巩固省级卫生城市创建成果，争取2015年创建成为国家卫生城市，让枣庄变得更加整洁。

(七)统筹推进城乡文明建设。扎实开展省级文明城市创建，力争尽快通过考核验收。着力抓好文明单位、文明社区、文明家庭、文明行业创建活动，弘扬文明新风。加强“四德”教育，开设市民道德讲堂、道德模范宣传栏，提升公民道德素质。强化诚信教育，建立诚信档案，惩治失信主体，增进政务诚信、商务诚信和社会诚信。繁荣发展文化事业，开展榴花文艺奖评选，鼓励创办庄户剧团、社区艺术团。建好用好图书馆、文化馆、博物馆、展览馆、美术馆、体育馆和城乡小型文体广场，合理布局一批电子阅报亭、公益广告栏，推动机关事业单位公共绿地、文体设施向社会开放，丰富群众精神文化生活，让枣庄变得更加文明。

六、切实抓好民生保障和社会事业发展

民之所望，政之所向。市政府将把增进民生福祉放在更加突出的位置，进一步加大民生投入，大力发展各项社会事业，用心办好百件惠民实事，让群众得到更多看得见、摸得着的实惠。坚持以创业促就业。引导大学生自主创业、农民离土创业、成功人士返乡创业、民营企业二次创业，激活各类创业主体。年内新增城镇就业4.2万人，农村劳动力转移就业5.6万人。今年，全市将购买1000个公益性岗位和1000个大学生见习岗位，帮助特困家庭、双零家庭、下岗失业人员和高校毕业生就业。加大产业扶贫开发力度，鼓励创办农村特色二三产业，帮助更多的农民脱贫致富。进一步提高社会保障标准。城乡居民养老保险缴费补贴最高标准增至50元；城市低保提高到每人每月410元，农村低保提高到每人每年2455元；五保集中供养、分散供养标准分别提高到每人每年4230元和3100元；为80周岁以上享受低保的老年人增发高龄补贴。继续提高企业退休人员基本养老金、城乡居民基本医疗保险补助等标准。完善城市基础设施。新建改造供热管网46公里，新增集中供热面积300万平方米；加快建设街头游园、城市林荫道路和林荫停车场，新建城区小绿地28万平方米；下决心对19个老旧小区和92条背街小巷进行整治，实施路面整修、绿化亮化、违建拆除和管网疏通。规范提升社区放心粮油店25家，新增便民快餐示范店20家、便民快餐网点60个。提升公共服务水平。新建改建幼儿园21所、中小学校舍15万平方米、学生食堂50处；实施市特教学校迁建工程；新建40个留守儿童快乐成长活动站。选派500名优秀医生进农村、入社区开展诊疗服务；免费为1500名贫困白内障患者实施复明手术。启动市社会福利养老服务中心和市残疾人康复中心建设，新建改建社区老年人日间照料中心30处、农村幸福院60处，升级改造乡镇敬老院30处。创新社会治理。深化平安枣庄建设，有效防范和严厉打击各类违法犯罪活动。全面落实安全生产责任，强化重点行业领域安全监管、隐患排查治理和应急处置，确保安全生产形势持续稳定。抓好食品药品安全监管，确保群众饮食用药安全。完善粮食储备体系，稳定基本生活品供应价格。综合治理出生人口性别比。扎实开展双拥共建。同时，推动民族宗教、外事侨务、知识产权、档案史志、妇女儿童、残疾人、红十字、老龄、人防、地震、气象等各项事业全面发展。

七、进一步加强政府自身建设

打好改革攻坚战、实现群众新期盼，把枣庄发展得更好、建设得更美，是对各级政府的重大考验。我们将牢记全市人民的重托，按照为民、务实、高效、廉洁的要求，抓好政府自身建设。

(一)加快指导转变。各级政府要以脱胎换骨的勇气，切实在发展理念、发展定位、发展动力、发展途径、考核导向上实现新转变，坚决摒弃盲目的、不切实际的增长方式和发展观，依靠改革推动、创新驱动、开放带动，增创转型优势、增添发展活力。坚持“差异定位、特色发展、分类考核”，引导各级政府按照主体功能区的理念，发挥优势、统筹谋划、突出重点，努力实现科学发展、协调发展。改进和完善政绩评价体系，提高资源消耗、生态环境、科技创新、社会保障、安全生产等指标的权重，以考核导向的转变促进发展方式的转变。

(二)践行群众路线。讲认真、重实效，扎实开展群众路线教育实践活动，切实解决群众反映强烈的“四风”问题，进一步密切与群众的血肉联系。带着感情下基层，灶前床头访贫苦，掏心窝子办实事，把民生工作做到老百姓的心坎上。畅通依法有序的民意表达渠道，通过市长热线、市长信箱、政风行风热线等，多领域开展群众满意度测评、社情民意调查，广泛听取群众的意见和呼声。坚持领导干部定期公开接访，加快网上信访平台建设，有效解决群众关切的热点问题，确保群众合理诉求得到及时回应、合法权益得到切实维护。

(三)强化依法行政。推动政府管理与服务创新，注重运用法治思维深化改革、化解矛盾、推动发展。健全集体议事、专家咨询、公开听证、合法性审查等制度，确保政府每一项重大决策都遵循法治的轨道、经得起实践和历史检验。实行“权力清单”和“责任清单”制度，严格行政执法程序，规范行政权力运行，做好行政复议应诉工作，以政府依法行政带动全社会知法守法。完善政务公开制度，利用政府公报、门户网站、发布会等形式，推进决策公开、服务公开、结果公开。认真执行人大及其常委会的决议、决定，扎实做好人大代表建议和政协提案办理工作。拓宽民主党派、工商联和无党派人士参政议政的渠道，引导社会各界为经济社会发展建言献策。

(四)恪守廉洁奉公。认真落实“一岗双责”，严格履行党风廉政建设责任制，将防治腐败的要求贯穿深化改革全过程。突出政府采购、土地出让、工程招投标、项目审批等重点领域、关键环节，加大行政监察、审计监督、财政监管力度，确保行政权力运行到哪里、监督就延伸到哪里。持续开展“百千百”民主评议和“庸懒散”专项治理，认真解决征地拆迁、劳动争议、涉法涉诉等领域存在的突出问题，切实纠正损害群众利益的不正之风。大力倡导勤俭节约，坚决反对奢侈浪费，严格落实中央“八项规定”，带头执行公务接待、公务用车、办公用房标准，年内“三公”经费再压减5%以上，以廉政建设的新成效取信于民。

各位代表!改革发展任重道远，转型振兴催人奋进。让我们紧密团结在以习近平同志为总书记的党中央周围，在市委的坚强领导下，凝聚起攻坚克难、干事创业的强大合力，加快建设转型升级和经济文化融合发展高地，努力谱写枣庄人民幸福生活新篇章!
